# A paper-based microfluidic platform with shape-memory-polymer-actuated fluid valves for automated multi-step immunoassays

**DOI:** 10.1038/s41378-019-0091-0

**Published:** 2019-09-23

**Authors:** Hao Fu, Pengfei Song, Qiyang Wu, Chen Zhao, Peng Pan, Xiao Li, Nicole Y. K. Li-Jessen, Xinyu Liu

**Affiliations:** 1 Department of Mechanical and Industrial Engineering, University of Toronto, Toronto, ON M5S 3G8 Canada; 2 Department of Mechanical Engineering, McGill University, Montreal, QC H3A 0C3 Canada; 3 Department of Electrical and Electronic Engineering, Xi’an Jiaotong-Liverpool University, 215123 Suzhou, Jiangsu China; 4 School of Communication Sciences and Disorders, McGill University, Montreal, QC H3A 1G1 Canada; 5 Institute of Biomaterials and Biomedical Engineering, University of Toronto, Toronto, ON M5S 3G9 Canada; 6 Present Address: Department of Chemistry, Stanford University, Stanford, CA 94305 USA

**Keywords:** Engineering, Chemistry

## Abstract

Smart fluid manipulation with automatically controlled paper valves will enable automated and multi-step immunoassays on paper-based microfluidic devices. In this work, we present an integrated paper-based microfluidic platform with shape-memory polymer (SMP)-actuated fluid valves capable of automated colorimetric enzyme-linked immunosorbent assays (ELISAs). A single-layer microfluidic paper-based analytical device (μPAD) was designed to store all the reagents on the chip, and sequentially transfer reagents to a paper test zone following a specific ELISA protocol through automatic fluidic flow control by the multiple SMP-actuated valves. The actuation of a paper valve was based on the thermally responsive, duel-state shape transformation of a SMP sheet attached to the root of a paper cantilever beam for driving a hydrophilic paper bridge to connect and disconnect two paper channels. A portable colorimetric reader was developed to control the on-chip valve operations, quantify the colorimetric signal output, display the assay result, and wirelessly transmit the data to a smart phone for the application of telemedicine. Reliable operations of the paper valve and the entire μPAD were demonstrated with success rates of 97% and 93%, respectively. A detection mechanism for valve malfunction was designed and confirmed effective to identify any mal-operation of individual valves, thus rendering our platform reliable in real assays. For device calibration, we conducted direct ELISAs of rabbit IgG in phosphate-buffered saline (PBS), and achieved a low limit of detection (LOD) of 27 pM (comparable to that of standard and paper-based ELISAs). In order to demonstrate the clinical application of our multi-step immunoassay platform, we also conducted sandwich ELISAs to quantify the protein level of an inflammatory cytokine, namely tumor necrosis factor (TNF)-α, in surgically injured laryngeal tissues of rats. The protein levels of TNF-α were shown similar between the conventional and μPAD ELISAs.

## Microfluidics: Paper valves open doors to new designs

Researchers in Canada have improved the design of paper-based analytical devices by developing paper valves which make it possible to carry out fully-automated multi-step analyses. Previous efforts to incorporate valves have been based on electromagnets or compressed sponges, making them too cumbersome or complicated for reliable use in paper-based analytical devices. To overcome this, a team led by Xinyu Liu of the University of Toronto turned to polymers that reversibly change shape in response to temperature. They combined these with a heating element to produce thermally-activated paper valves. Test devices designed to carry out a multi-step ELISA analysis produced results comparable to a standard commercial ELISA kit. The valve design can be used to create devices for other multi-step diagnostic assays which can be used in contexts where lab equipment and skilled personnel are unavailable.

## Introduction

Point-of-care testing (POCT) is designed for rapid diagnostic assays with satisfactory accuracy and sensitivity, low sample/reagent consumptions, and excellent cost efficiency. POCT has enabled effective healthcare in resource-limited settings, and has supplemented or replaced the conventional diagnostics of existing healthcare systems. Microfluidic paper-based analytical devices (μPADs) represent one of the most promising platform technologies for POC diagnostics, in which paper substrates are employed to bring various merits to analytical tests^1^. Many types of bioassays have been implemented on the μPADs by colorimetry^2–4^, fluorometry^5^, electrochemistry^6^, and electrochemiluminescence^7^ for detection of proteins based on binding of reporters (e.g., ionized color dyes and enzyme-conjugated antibodies) to target analytes. Among a diverse range of bioassays, the enzyme-linked immunosorbent assay (ELISA) is a gold standard of protein detection in clinical samples associated with diseases^3,4^. However, in most of the existing μPAD designs running ELISAs^3,8^, the multi-step assays were performed manually. The assay process involves human operations such as repeated pipetting of samples/reagents and quantification of the assay results using imaging devices such as scanner, camera, and microscope. Often, the user needs to operate the μPAD by following a specific protocol. Thus, the inability of these μPADs to autonomously carry out the entire ELISA process limits, to some extent, their applications to certain diagnostic scenarios where some tests would need to be conducted in a ‘sample-in-result-out’ (SIAO) fashion.

Although the capillary wicking in a porous paper substrate of μPADs eliminates the requirement of external pumps for driving fluids to perform assays, controlled fluid manipulation in porous paper channels is not as straightforward as in the conventional hollow microfluidic channels. A variety of fluid manipulation strategies on μPADs have been developed for achieving on-chip fluid control at certain levels, including mechanical valves^9–12^, channel-geometry-based fluid regulation^13,14^, fluidic diodes^15–17^, dielectric electrowetting^18^, dissolvable bridges^19,20^, meltable wax valves^21,22^, porous shunt^23^, paper carving^24^, selectively permeable barrier^25^, and electrostatic control^26^. Benefitting from these fluid manipulation strategies, human operation is no longer a necessity for fluid regulation on a μPAD. Among these fluid manipulation methods, the mechanical valves are straightforward in terms of device design, and primarily rely on the control of connection and disconnection between channels. For instance, a paper cantilever beam was operated manually to control the connection and disconnection of two channels^9^. Sliding operation can bring channels in different paper layers into contact to transfer fluids and run multi-step assays^4,10,27^. Push-button valves were designed for manual compression to bridge a gap between two channels in different layers of a μPAD for fluid transport^11^. Besides valve designs in devices with hydrophilic paper channels, μPADs with embossed hollow channels also involved a valving mechanism through folding and unfolding of the paper substrate to turn on and off fluid flows in the hollow channels^12^. One common drawback of the aforementioned valve designs is the requirement of manual operations.

Automated operations of mechanical valves on μPADs were also demonstrated, making autonomous on-chip assays possible. We previously reported a magnetic timing valve for timed fluid control on μPADs^28^. This design functionalized a paper cantilever beam with magnetic nanoparticles, and used an electromagnet to actuate the paper beam for one-way connection or disconnection of a paper channel. The operation of each paper beam valve, however, requires a separate electromagnet. For a μPAD with multiple magnetic valves, the required electromagnets need to be separated from each other with a sufficient spacing to avoid mal-manipulation of paper valves caused by magnetic cross-talks. Also, the same electromagnet can only activate or deactivate its corresponding valve, i.e., one-way operation. On the other hand, Thuo and co-workers proposed a two-way operation design of magnetic paper valves^29^. Hard magnets were patterned on one end of a paper cantilever valve through impregnation of hard magnetic polymer, allowing the valve to be magnetically attracted or repelled by a magnet and thus operate at three different positions, i.e., the neutral, on, and off positions. However, similar to our previous design^28^, each valve still needs an electromagnet for actuation, and the relatively large size of the electromagnet and the required large spacing of adjacent electromagnets (for avoiding valve cross-actuation) limit the integration density of these valves on a μPAD. Yager and co-workers demonstrated a valve activation method on μPADs with compressed sponges as localized actuators in an integrated diagnostic toolkit^30^, and performed ELISAs for SIAO testing. However, the device includes many moving parts (e.g., test strips, sponge-based valves, and glass fiber actuation channels), making its usage less straightforward and its manufacturing potentially challenging. For continuous testing of multiple samples, the loading of a new μPAD may need assembly of a paper strip and its moving parts, thus lowering the operation efficiency. Furthermore, the toolkit only provides qualitative diagnostic answers directly, and subsequent analysis by a professional is required for obtaining quantitative results.

In this paper, we present a new design of thermally-activated paper valves that enables the development of a fully-automated paper-based microfluidic device for performing colorimetric ELISAs in a SIAO fashion. The thermally responsive shape-memory polymer (SMP) was used, for the first time, to actuate a paper cantilever beam on a μPAD for activation and deactivation of a paper valve. A unique feature of our valve design is that the same SMP actuator is capable of both turning on and off a valve, which is based on the due states (temporary and permanent shapes) of the SMP material. We designed a μPAD that stores all the required reagents on a chip and sequentially transports individual reagents to a test zone of the device to enable a multi-step ELISA. We also developed a portable colorimetric reader to control the on-chip paper valves for automatically conducting an ELISA and to quantify the colorimetric output. To run an assay, a user only needs to mount a µPAD onto the platform, add the sample and reagent-transferring buffer to the µPAD, and wait until the assay is completed and the result is displayed on the device screen. The platform typically completes a direct ELISA within 55 min. We also proposed a novel self-checking mechanism for reliably monitoring valve malfunction, which is based on detection of the light-transmittance differences of the test zone in wet versus dry states. By using this platform, we performed direct ELISA of rabbit IgG in phosphate-buffered saline (PBS) and sandwich ELISA of tumor necrosis factor (TNF)-α, an inflammatory cytokine marker, obtained from rat laryngeal tissue samples, and achieved high analytical performance that is comparable to that of standard ELISA performed on a 96-well plate.

## Results and discussion

### The µPAD with SMP-actuated valves

**Fig. 1:**
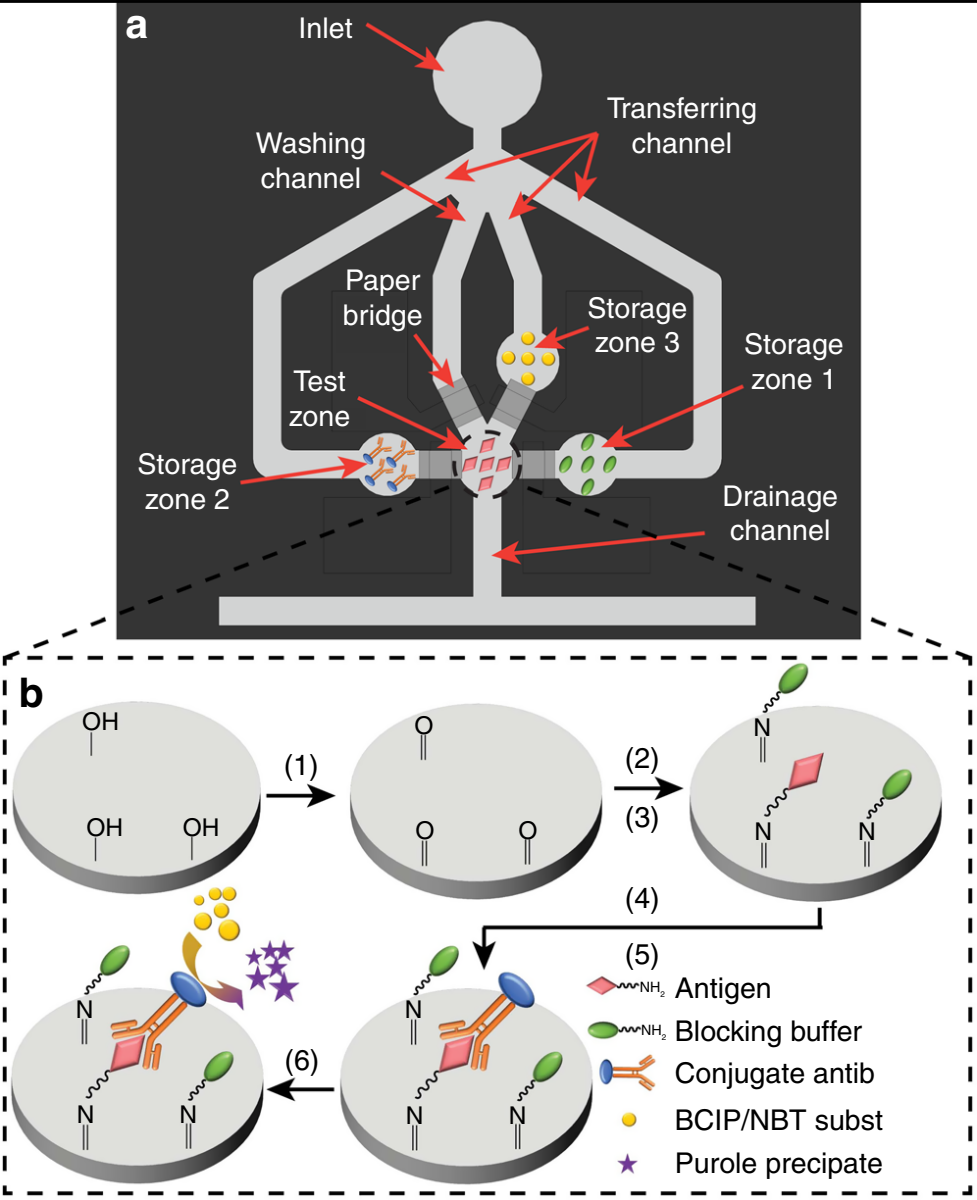
Schematic diagrams of the μPAD design and the direct ELISA protocol. **a** Schematic top view of a µPAD integrating four SMP-actuated valves (the valves are attached to the back of the µPAD) and **b** The schematic protocol of a direct ELISA that can be performed in the test zone of the µPAD. The protocol of a direct ELISA is carried out in six steps: (1) modifying the paper test zone surface with aldehyde groups using potassium periodate (KIO_4_); (2) immobilizing antigens on the modified paper through covalent bonding; (3) blocking the test zone surface with blocking buffer; (4) labeling antigens with enzyme-conjugated antibodies; (5) washing away unbound antibodies; and (6) adding enzyme substrate for colorimetric signal production

Figure 1a schematically illustrates the design of a µPAD with four SMP-actuated paper valves to run a direct ELISA. All reagents required by the ELISA are stored (in a dry form) in the storage zones (6 mm in diameter) that are all connected, through the paper valves, to a central test zone (6 mm in diameter). To carry out a test, a sample solution is first added to the test zone and five drops (total volume: 250 µL) of 1 × PBS buffer to the device inlet with a syringe. Then, individual reagents are transferred to the test zone sequentially in a pre-defined order, and different valves are activated accordingly to allow the PBS to transfer the reagents to the test zone. The SMP-actuated valves are automatically controlled by a colorimetric reader (on which the µPAD is operated) to switch on and off, and the reader determines the sequence and timing of reagent transfers based on a pre-programmed ELISA protocol.

Thermally responsible SMPs have been used to fabricate actuators of origami robots to achieve localized joint rotation and three-dimensional folding/unfolding^31,32^. A piece of thermally responsive SMP can deform from its permanent shape to a temporary shape when heated to a temperature above its switching transition temperature (*T*_trans_), and then maintain its temporary shape when cooled down to a temperature below *T*_trans_. When heated up again above *T*_trans_, the SMP will transform back to its permanent shape. Based on this dual-state property, we introduced the thermally responsive SMP in our device design for two-way actuation of the paper valve, thus enabling automatic fluid control on a μPAD for running multi-step assays.

**Fig. 2:**
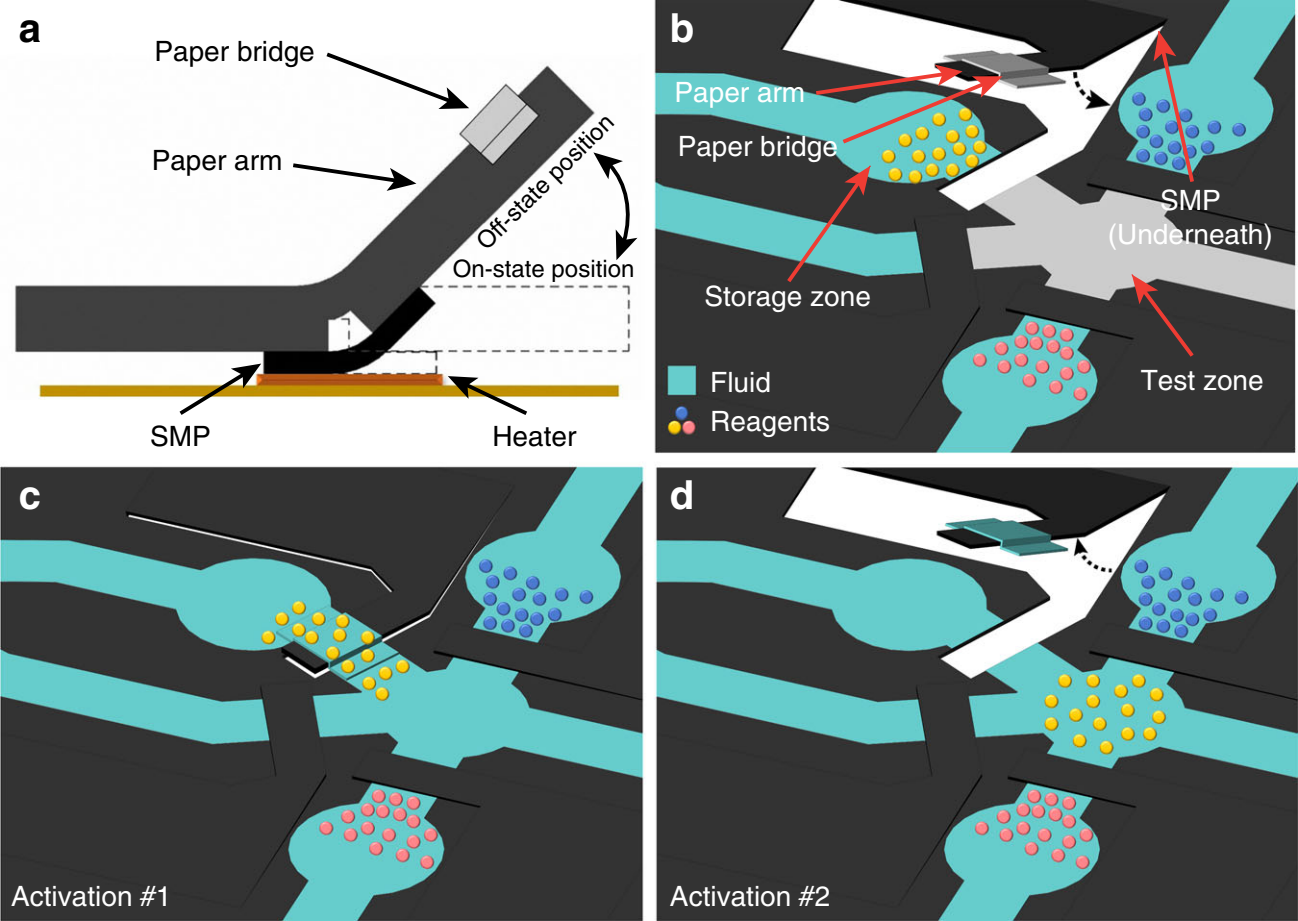
Principle and operation of the SMP-actuated valve on the µPAD. **a**, **b** Side and oblique views of a paper cantilever arm that is laser-cut out of a single-layer μPAD and attached with a SMP-actuated valve at its root. The initial state of a valve is bent up. **c** The SMP is first activated to reach its temporary flat shape (“activation #1”), which bends down the paper arm and makes the paper bridge connect the reagent storage zone and the test zone for reagent transfer. **d** The SMP is then activated again to recover its permanent curved shape (“activation #2”), which disconnects the reagent storage zone and the test zone and stops the transfer process. The dashed lines indicate the actuation paths of the paper cantilever beams for activation #1 and #2

As schematically shown in Fig. 2a, a sheet of polyolefin (PO; *T*_trans_ = 95 °C) with a curved permanent shape was attached to the root of a paper cantilever arm (which is laser-cut out of a single-layer μPAD), and used as a bending SMP actuator. The initial bending angle of the paper arm was 30°. A copper heating resistor, patterned on a printed circuit board (PCB), was arranged underneath the SMP to activate the valve through joule heating^31^. When heated above 95 °C, the SMP lowers the paper beam to its on-state position (Fig. 2c; which is called “activation #1”), and can maintain this position even the heating is removed. When heated above 95 °C again, the SMP raises the paper arm back to its off-state position (Fig. 2d; “activation #2”). A hydrophilic paper bridge attached to the end of the paper arm connects the reagent storage zone and the test zone when the valve is turned on.

We experimentally determined in-plane size of the PO sheet and its heating resistor underneath to be 6 mm × 12 mm, which was large enough to ensure efficient heating and activation of the PO sheet. With the selected in-plane dimension of the PO sheet, we also compared the operation success rate of the paper valves made from three different types of commercially available PO sheets, and eventually chose the one (RNF-100 1″ × 4′ BLK, TE Connectivity) with a thickness of 0.89 mm as it provided the highest activation success rate and appropriate response time (Table S1). With the final designs of the SMP valve and the heating resistor, the minimal heating time required for activations #1 and #2 was measured to be 22.7 ± 3.7 s (*n* = 15) and 24.4 ± 5.3 s (*n* = 15), respectively (Table S1). Note both time periods were measured with the SMP valve initially at room temperature. To reliably activate the SMP valve to turn on and off, we set a fixed heating time at 35 s for both activation steps of #1 and #2. During operation of the SMP valve, no temperature rise was measured in the paper test zone.

### Integration of the μPAD with a colorimetric reader

**Fig. 3:**
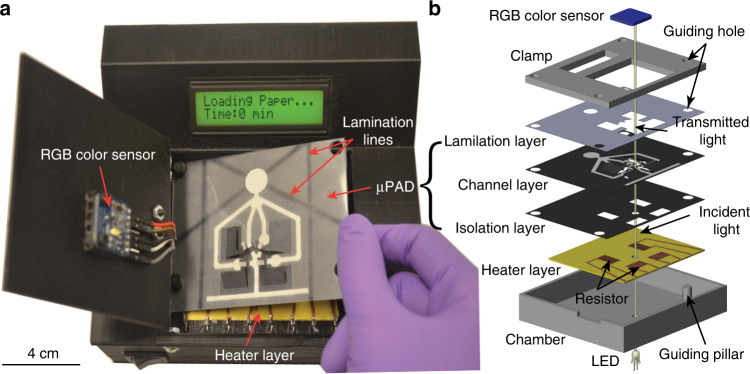
Integration of the μPAD with a colorimetric reader. **a** Photograph of the colorimetric reader that accommodates a µPAD for activation of the valves and readout of the colorimetric signal. **b** Exploded view of the combined architecture of the µPAD and the operation cell of the colorimetric reader

To automatically run an ELISA and measure its colorimetric output, we developed a portable colorimetric reader (Fig. 3a) to host the µPAD. The reader includes three major parts: (i) an operation cell for automatic operation of the on-chip paper valves and accurate measurement of the output signal, (ii) a microcontroller circuit to control the operation cell, and (iii) a Bluetooth module for data transmission to a cellphone or a computer. In the operation cell, a PCB with patterned copper heating resistors is arranged underneath the µPAD to activate the SMP actuators. The heating resistors were patterned by wet etching of the copper layer of a copper-coated polyimide sheet. For maximizing the heating efficiency, the copper traces of each heating resistor were patterned in a serpentine shape of 0.5 mm wide^31^, and the overall size of the resistor was designed to be the same (6 mm × 12 mm) as the SMP sheet size of the valve. The resistance of the heating resistor was 0.3 Ω, and heating current was 2 A. Each resistor was switched on and off by a 1.2 W transistor controlled by the microcontroller. To integrate with the colorimetric reader, an isolation layer (made from wax-impregnated paper) with laser-cut openings (Fig. 3b) was attached to the bottom surface of the paper channel layer with double-sides tapes, which only exposes SMP sheets of the valves to the copper heater and avoids fluid leakage from the paper channel layer to the heating PCB. A plastic lamination layer (Fig. 3b) was attached on the top surface of the paper channel layer to accelerate fluid flowing rate and prevent the evaporation of the buffer. The three layers of the µPAD were assembled by thermal lamination.

To quantify the colorimetric readout, a white LED (*λ*_max_ = 550 nm) on the bottom of the operation cell was used to illuminate the test zone of the µPAD when the last signal amplification step of the colorimetric ELISA was completed. A red-blue-green (RGB) color sensor (TCS34725, Adafruit) was arranged on the other side of the test zone to measure the light transmission through the test zone. The centers of the LED, the RGB sensor, and the test zone were aligned coaxially. A higher colorimetric signal level of the ELISA causes a lower level of light transmission through the test zone. The RGB sensor directly provides a digital electrical signal to the microcontroller, and the mean grayscale intensity [calculated from the RGB intensity from the sensor by taking the average of the three channel intensities: grayscale = (R + G + B)/3] is displayed on the LCD screen as the final result. The test results can also be transmitted to a cellphone or computer through Bluetooth communication. By pre-programming a specific ELISA protocol (i.e., timings of valve operations and colorimetric signal readout) in the microcontroller, and automatic ELISA can be achieved on the platform in a SIAO fashion. To run a test, a user only needs to load a µPAD to the reader, add a drop of sample to the test zone and several drops (~250 µL) of PBS buffer to the device inlet, then close the door and wait for a period of time before the result is displayed.

Figure S1 illustrates the graphic user interface (GUI) of Bluetooth communication application (APP) on an Android-based cellphone (Xperia™ Z, SONY). The Bluetooth module can transmit assay results to the cellphone, and the cellphone APP can display the data graph and further transmit the results to a remote site through text message. The cellphone APP can control the Bluetooth port of the platform, trigger the automated assays, and receive the data via Bluetooth transmission.

### Determination of the operation parameters

Before calibrating the device performance, we first experimentally determined two major operation parameters, namely, (i) the reagent transfer time: the necessary time required for transferring an on-chip stored reagent from its storage zone to the test zone (i.e., the on-state period of the valve) under continuous flow of PBS buffer; and (ii) the test zone washing time: the time required for removing unbound antibodies that are added to the test zone to bind with the target antigen in direct or indirect ELISA under continuous PBS flow. To determine the reagent transfer time, we performed reagent transfer experiments on the µPAD using fluorophore-conjugated rabbit IgG antibody (anti-rabbit IgG). First, we spotted 3 μL of fluorescein isothiocyanate (FITC)-conjugated anti-rabbit IgG solution (0.1 mg/mL) and 3 μL of blocking buffer solution [0.5% (v/v) Tween-20 and 10% (w/v) BSA in PBS] on the storage zones, and waited for 10 min at room temperature to dry the test zone. Then, we added ~250 μL of PBS to the inlet using a syringe. Once the PBS flow passed the storage zone and reached the paper valve (which was initially in its off-state), the valve was switched on to allow the PBS flow to transfer the FITC-conjugated anti-rabbit IgG to the test zone. The regent transfer time was counted as the time period during which the valve was on and the reagent-carrying PBS solution was flowing through the test zone. Once the valve was turned off, the test zone was incubated at ambient environment for 10 min. Finally, the FITC fluorescence intensity of the test zone, which corresponded to the amount of anti-rabbit IgG transferred to the test zone, was quantified by a fluorescence microscope (SZX-16, Olympus; excitation: 490 nm, and emission: 520 nm) with a cooled fluorescence camera (EXi Blue, QImaging). We found that the measured FITC intensity plateaued after a transfer time of 45 s (Fig. S2a). Thus, after the 35 s heating for activation #1 was completed, we set a 30 s waiting time before starting activation #2 to cool down the SMP valve to room temperature. As the heating time for activation #2 is 24.4 ± 5.3 s (before which the valve was on), the total reagent transfer time (average: 30 + 24.4 = 54.4 s) was found to be sufficient to achieve the maximum transfer efficiency.

With regard to the test zone washing time, we added 3 μL of FITC-conjugated anti-rabbit IgG directly to a test zone (without surface functionalization), incubated it for 1 min, and used the washing channel (Fig. 1a) to wash the test zone by continuous PBS flow from the inlet. Since the test zone was not aldehyde-functionalized, the added anti-rabbit IgG only formed weak physical bonding (similar to non-specific binding in an ELISA) with the surfaces of cellulose microfibers in the test zone, and can, in principle, be completely washed off under thorough perfusion. We quantified the residual FITC intensities of the test zones that had been washed for four different time lengths (30, 90, 150, and 210 s). From Fig. S2b, once can see that after 150 s washing, more than 90% of the non-specific binding in the test zone was removed. Accordingly, for activating the washing valve, the waiting time between activation #1 and #2 was set to be 130 s (average time for washing: 130 + 24.4 = 154.4 s).

### Success rate of valve operation and self-checking mechanism for valve malfunction

Video S1 shows the loading of a μPAD onto the colorimetric reader, and Video S2 illustrates the operation of four SMP-actuated valves on a μPAD for direct ELISA. In Video S2, colored dyes were used to mimic the ELISA reagents and were pre-stored in the reagent storage zones for visualization of the reagent transfer. Note that the door of the colorimetric reader, which should be always closed during a real assay, was opened for taking Video S2. We operated 100 μPADs of this kind (which include 400 SMP-actuated valves in total) to quantify the success rates of valve operation and device operation. Among the 400 valves, 388 valves operated normally, corresponding a success rate of valve operation at 97%. We counted the operation of a μPAD as a failure if any of its four valves malfunctioned, and obtained a success rate of device operation at 93% (*n* = 100). To further improve the device operation success rate, other SMP materials with different thicknesses could be investigated.

Despite the high success rate of valve operation, it is still highly desired to detect the malfunction of a specific valve in the practical use of a μPAD. To this end, we established a self-checking mechanism for valve malfunction through detecting the wetting condition of the test zone. We used the LED and RGB sensor pair to measure the transparency because a wet paper is more transparent than a dry paper. If a valve fails to switch on (activation #1 fails), no PBS buffer will be transferred to the test zone and a lower transparency of the test zone will be measured. Similarly, the failure of a valve during activation #2 (switching off) will cause the PBS still to flow through the test zone, thus induce a test zone transparency higher, after 1-min waiting, than normal. Thus, by detecting the light transmittance through the test zone right after a valve was switched on (activation #1) or 1 min after a valve was switched off (activation #2), the colorimetric reader was capable of detecting any valve malfunction with a detection success rate of 100%.

**Fig. 4:**
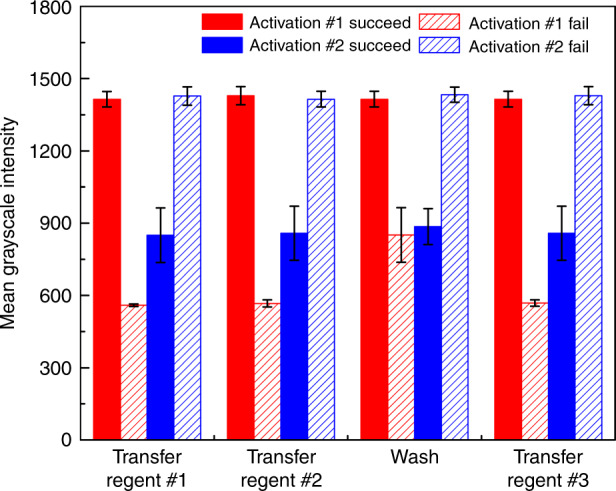
Light-transmittance signals (*n* = 5) measured from the test zone at each step of the direct ELISA (Fig. 1b), right after the valve is in activation #1 and 1 min after the valve is in activation #2. Reagents #1, #2, and #3 are the blocking buffer, the enzyme-conjugated antibody, and the enzyme substrate. When the valve activation fails to work after each step, the measured light-transmittance signals of the test zone are different from the normal state based on the unusual wetting conditions, enabling the detection of the valve malfunction

For the direct ELISA protocol (Fig. 1b), we carried out reagent transfer experiments and measured the mean grayscale intensities of the light transmitted through the test zone before and after activations #1 and #2 for each valve of the μPAD (results shown in Fig. 4). For activation #1 of the valve, the grayscale intensities were measured right before the activation #1 was initiated (i.e., heating resistor was turned on) and 10 s after the 35 s heating for activation #1 was completed. For activation #2 of the valve, the grayscale intensities were measured right before the activation #2 was initiated and 1 min after the 35 s heating for activation #2 was completed. From Fig. 4, one can see that the grayscale intensity varied significantly from its normal value if a valve failed during activations #1 and #2, allowing the reliable detection of any valve malfunction during an assay. Once a valve malfunction is detected, the colorimetric reader stops the ongoing assay and reminds the user of the failure. As we obtained a high device operation success rate of 93%, statistically only 7% of μPADs may fail during operation. This 7% of failed devices were all detected with this self-checking mechanism.

### Automatic direct ELISA of rabbit IgG antigen in PBS

As a proof of demonstration, we carried out automated direct ELISA for the detection of rabbit IgG on our platform. Before each experiment, the following reagents were stored on the μPAD: (i) 3 μL of blocking buffer of 0.5% (v/v) Tween-20 and 10% (w/v) BSA in PBS, (ii) 3 μL of PBS solution of the alkaline phosphatase (ALP)-conjugated IgG antibodies (0.1 mg/mL), and (iii) 3 μL of 5-bromo-4-chloro-3-indolyl phosphate and nitro blue tetrazolium substrate (4.59 mM BCIP, 3.67 mM NBT, 50 mM MgCl_2_ in 1 M Tris buffer, pH 9.5). Then, we waited for 10 min at room temperature to dry the storage zones. If long-time storage of the prepared μPAD is needed before an assay, one could storage the device in an air-tight bag with desiccator and also added protein stabilizer to prevent the degradation of the stored reagents^8^.

To run a test, we mounted a µPAD onto the colorimetric reader, added 3 μL of the rabbit IgG solution to the test zone and ~250 μL of PBS washing buffer to the µPAD inlet, and then initiated the assay. The platform was pre-programmed to automatically control individual valves and carry out the following steps of the assay. (i) The blocking buffer was transferred to the test zone 3 min after the assay was started (the 3-min waiting time allows the washing buffer to travel from the inlet to the individual valves), and incubated for 10 min. (ii) The ALP-conjugated antibody was then transferred to the test zone for labeling the immobilized antigens, and incubated for 1 min. (iii) The valve connecting the washing channel and the test zone was switched on to wash the test zone for 2.5 min and remove any unbound antibodies. After the washing valve was switched off, the operation paused for 10 min to drain the test zone. (iv) The BCIP/NBT substrate was transferred the test zone, and incubated for 30 min for signal amplification. (v) The pair of LED and the RGB color sensor was finally initiated for quantifying the colorimetric signal. Note that all the assay steps were performed at room temperature. The assay result was expressed as the mean grayscale intensity calculated from the raw RGB outputs from the RGB color sensor. The result was directly displayed on the LCD of the colorimetric reader for data recording. During the assay, the self-checking mechanism for valve malfunction constantly monitored the valve operations. Once a valve malfunction occurred, the platform reported an error message on the LCD and reminded the user to replace the µPAD.

**Fig. 5:**
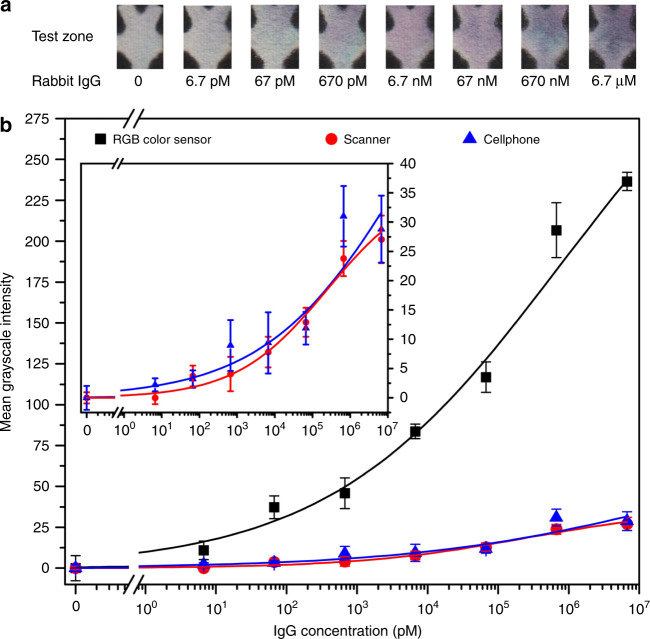
Results of direct ELISA on the μPAD for detection of rabbit IgG in PBS. **a** Photographs of test zones at different IgG concentrations. **b** Calibration curves of the mean grayscale intensity signal versus the IgG concentration on the test zone (*n* = 5). Inset: amplification of the low mean grayscale intensity range (from 0 to 40) of the calibration curves. Signals were measure from the same test zones by the RGB color sensor (*R*^2^ = 0.993), a desktop scanner (*R*^2^ = 0.970), and a cellphone camera (*R*^2^ = 0.894). The RGB color sensor provides higher intensity signals than the scanner and cellphone

We carried out direct ELISA of rabbit IgG in 10-fold dilutions (6.7 pM to 6.7 μM), and the calibration data of our platform was shown in Fig. 5a. We also captured the test zone image after each assay with a desktop scanner (CanoScan LiDE 210, CANON Inc., setting: color photo scanning, 300-dpi resolution) and a cellphone camera (Xperia™ Z, SONY Electronics Inc., image size: 4128 × 3096 pixels). For the cellphone, we took the test zone images inside a mini photo studio (Mini Portable Photo Studio Shooting Tent, JHS-TECH) with uniform illumination, which minimized the illumination variations of the ambient environment. We measured the mean grayscale intensities of all the test zones using ImageJ by the same average method for the colorimetric reader. All the data sets were fitted into sigmoidal curves (*s*-curves in Fig. 5b) using the Hill equation^3^ (Eq. S1), from which the limit of detection (LOD) and the coefficient of determination (COD; denoted as *R*^2^) for each *s*-curve were calculated.

The calculated LODs and CODs are LOD = 27 pM and *R*^2^ = 0.993 for the RGB color sensor, LOD = 255 pM and *R*^2^ = 0.970 for the scanner, and LOD = 836 pM and *R*^2^ = 0.894 for the cellphone. The LOD of our platform for rabbit IgG detection is comparable to that (18 pM) of the previously reported paper-based ELISA^3^. Despite the relatively uniform illumination provided by the mini photo studio, the mean grayscale intensities of the test zones measured by the cellphone camera were still subject to larger variations because of the ISO self-adjustment of the cellphone camera when taking a photo. Therefore, the cellphone provided the lowest signal-to-noise ratio (SNR; which is defined as the ratio of the average to the standard deviation of the intensity data at each concentration) among the three colorimetric measurement methods (Fig. S3), and thus the highest LOD and the lowest COD. The scanner employed uniform scanning illumination in an enclosed flatbed, making the SNR of its calibration curve higher (average: 3.76; Fig. S3), LOD lower, and COD higher than those of the cellphone. Compared to the desktop scanner and the cellphone, the RGB color sensor provided the highest SNR (average: 14.07; Fig. S3), the lowest LOD, and the highest COD.

The superior performance of our colorimetric reader for colorimetric measurement was due to the well-controlled illumination environment of our colorimetric reader, and more importantly, due to the transmission-based measurement mode of the colorimetric signal of the test zone. The scanner and the cellphone camera are both based on light reflection on the paper surface of the test zone, and can thus only quantify the color change on the test zone surface. In contrast, our colorimetric reader quantifies the transmitted light through the entire paper thickness of the test zone, which reflects the total color change of the test zone along its thickness. Thereby, the RGB sensor provided the highest SNR, the lowest LOD, and the highest COD. From the three calibration curves in Fig. 5b, one can also find that the RGB sensor provided the highest sensitivity among all the three methods.

### Autonomous sandwich ELISA for animal tissue samples

Sandwich ELISA is a popular protocol with high sensitivity and specificity, and is more widely used for analyzing real samples with a complex protein background than direct ELISA. We also carried out sandwich ELISA of TNF-α in protein extraction solutions from rat laryngeal tissue, specifically vocal folds, with our platform. TNF-α is a cell signaling molecule that regulates the response of immune cells to injury, inflammation, and healing. Dysregulation of TNF-α has been associated with multiple physiological dysfunctions and diseases such as cancer^33^, Alzheimer's disease^34^, major depression^35^, and inflammatory bowel disease^36^. To demonstrate the potential clinical application of our platform, protein samples extracted from surgically injured rat vocal fold tissues^37^ were subjected to both conventional and μPAD-based ELISAs for evaluation.

**Fig. 6:**
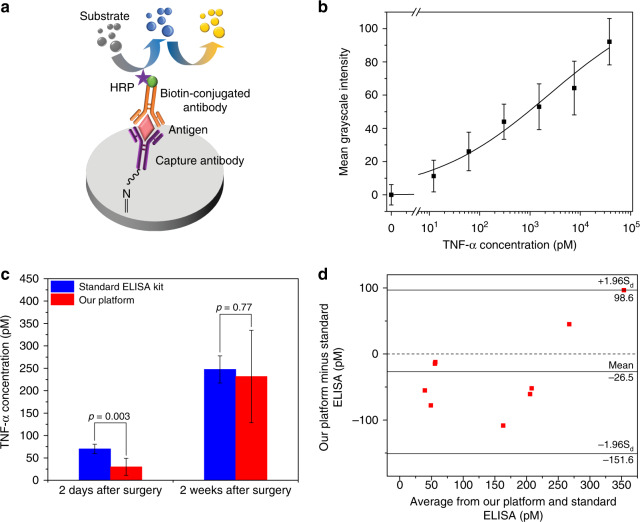
Sandwich P-ELISA for TNF-α in protein extractions from surgically injured rat vocal fold tissue tested by our platform and the standard ELISA. **a** Schematic of a sandwich ELISA on the µPAD. HRP substrates were catalyzed by HRP into blue products. The catalyzing reaction was stopped with sulfuric acid to produce yellow color. **b** Calibration curve of the mean grayscale intensity signal measured from our platform versus the TNF-α concentration (*n* = 5). **c** The comparison of testing data from our platform and the standard ELISA on extraction samples of rat vocal fold tissues two days and four weeks after surgery. **d** Bland–Altman analysis on the diagnostic methods based on our platform and the standard ELISA

We first performed sandwich ELISA on our platform for rat TNF-α spiked in 1 × PBS in five-fold dilutions (19 pM to 59 nM) to generate a calibration curve. Before each assay, 3 μL of anti-rat TNF-α in PBS (0.1 mg/mL) was added to the potassium periodate (KIO_4_)-functionalized test zone as the capture antibody, and dried at room temperature for immobilization. Then, 3 μL of the blocking buffer [0.5% (v/v) Tween-20 and 10% (w/v) BSA in PBS] was added to the test zone and dried at room temperature to fill all the vacant sites on the test zone. After that, 3 μL of rat TNF-α sample solution was spotted to the test zone for binding with the capture antibodies. 3 μL of 1:1 (v/v) mixture of biotin-conjugated anti-rat TNF-α (0.2 mg/mL in PBS; as the secondary antibody) and HRP streptavidin (0.2 mg/mL in PBS; to in situ label the secondary antibody with HRP) was stored in the storage zone #1 (Fig. 1a), 3 μL of HRP substrate (4 mM TMB in DMSO and 0.05 M phosphate-citrate buffer with trace amount of fresh 30% hydrogen peroxide, pH 5.0) in the storage zone #2, and 3 μL of stop solution (4 M sulfuric acid) in the storage zone #3. The sandwich ELISA was carried out with following steps. (i) The mixture of biotin-conjugated antibodies with HRP streptavidin was transferred to the test zone, and incubated for 1 min. (ii) the test zone was washed by PBS, and incubated for 10 min. (iii) The HRP substrate was transferred to the test zone, and incubated for 10 min. (iv) The stop solution was transferred to the test zone for the signal production, and incubated for 10 min. (v) Finally, the colorimetric signal was measured. Figure 6a shows the scheme of the sandwich ELISA on our μPAD. The measured results at different concentrations of TNF-α were fit into the Hill equation, as shown in Fig. 6b. The LOD of our platform for TNF-α detection was determined to be 22 pM. More thorough optimization of our sandwich ELISA protocol may further reduce the LOD of our platform. For confirming the effectiveness of pre-mixing the secondary antibody and HRP streptavidin and storing them in the same storage zone, we also carried out assays by sequentially adding the secondary antibody and the HRP streptavidin to the test zone as control experiments. We compared the results with these from our original protocol above through student *t*-test, and found no significant difference (*p* = 0.132, *n* = 7; Fig. S4) between the two data sets. This confirms the effectiveness of our original protocol.

Finally, we conducted sandwich ELISA for detection of TNF-α in protein extractions from injured rat vocal folds 2 days (*n* = 4) and 4 weeks (*n* = 5) following surgery^37^. We also benchmarked our platform through testing the same samples using a commercial ELISA kit (ab100785, Abcam) on a 96-well plate. Figure 6c shows the testing results from our platform (red bars) and the commercial ELISA kit (blue bars). For the sample group of 4 weeks after surgery, the TNF-α concentrations (231.6 ± 103.09 pM) measured by our platform reveal no significant difference (*p* = 0.77) from those obtained by the commercial ELISA kit (247.4 ± 30.3 pM). For the sample group of 2 days after surgery, their TNF-α concentrations (69.9 ± 10.4 pM) are close to the LOD (27 pM) of our platform; thus, the measurement results from our platform become significantly lower (*p* = 0.003) than these from the standard ELISA. These results show that our platform is capable of detecting animal cytokine proteins within the normal measurement range. From the standard ELISA results in Fig. 6c, one can also observe that the TNF-α concentration in the vocal fold tissue extractions increased from 69.9 pM 2 days after surgery to 247.4 pM 4 weeks after surgery, corresponding to the dual roles of TNF-α in the acute and later phases of wound healing^37,38^. To further evaluate the agreement of the two diagnostic methods based on our platform and the standard ELISA, we performed Bland–Altman analysis^39^ of the measurement results. As shown in Fig. 6d, the Bland–Altman analysis demonstrates a good agreement between the two methods with the mean difference (−26.5 pM) and limits of agreement (95% confidence interval: −151.6 to 98.6 pM). All of the sample points located within the 95% confidence interval around the mean. This result further demonstrates the potential use of our platform as a SIAO diagnostic platform for real diagnostic applications.

## Materials and methods

### Materials and reagents

Whatman No. 1 chromatography paper, bovine serum albumin (BSA), rabbit IgG, anti-rabbit IgG (alkaline phosphatase conjugated), anti-rabbit IgG (fluorescein isothiocyanate conjugated), 3,3′,5,5′-tetramethylbenzidine (TMB) (99%), BCIP®/NBT tablets, Tween® 20, 10 × phosphate buffered saline (PBS), and potassium periodate were purchased from Sigma-Aldrich (Oakville, ON, Canada), and used as received without further purification. Recombinant rat TNF-α, rat anti-TNF-α antibody, and horseradish peroxidase (HRP)-conjugated streptavidin were purchased from Abcam (Toronto, ON). Biotinylated anti-Rat TNF-α was purchased from BioLegend (San Diego, CA). The white LED and polyolefin (PO) were purchased from Digi-Key Cooperation (Thief River Falls, MN). The Arduino UNO microcontroller and the 16 × 2 LCD were purchased from RobotShop Inc. (Mirabel, QC). The Pyralux® (LF7062) copper-coated polymide film was obtained as sample from DuPont (Research Triangle Park, NC, USA). Ferric chloride was purchased for etching the copper from MG Chemicals (Burlington, ON, Canada). The RGB color sensor (TCS34725) was purchased from Adafruit (New York, NY, USA). The scotch plastic thermal laminating pouches were purchased from 3 M (Milton, ON, Canada).

### Fabrication and preparation of the µPAD

The channel layer and the isolation layer of the µPAD, both made from chromatography paper, were fabricated using wax printing and laser cutting^40^. To fabricate the channel layer, a Xerox 8570 inkjet printer was used to print solid wax (black) patterns on Whatman No. 1 chromatography paper to define the paper channels. The paper with solid wax printed on its surface was then placed on a hotplate at 120 °C for 30 s to melt the wax and form hydrophilic channels inside the paper. After that, paper cantilever beams were cut out of the channel layer using a CO_2_ laser cutter (VLS 2.30, Universal Laser Systems). The root of each paper cantilever was cut using dashed lines to form a foldable hinge, and the free end of each paper cantilever was cut to disconnect the test zone and a reagent storage zone (Fig. 2b). A hydrophilic paper bridge was cut out of soft tissue paper (Delicate Task Wipers, Kimwires) and attached to the free end of the paper cantilever valve. This paper bridge connects the test zone and the storage zone when the paper valve is driven to its on-state position (Fig. 2c). The isolation layer was constructed by fully impregnating Whatman No. 1 chromatography paper with solid wax (through wax printing and 120 °C baking) and then laser-cutting it to form 6 mm × 12 mm windows with their positions aligned with the ones of the heating resistors (on the heating PCB) and the SMP actuators (on the channel layer). The channel layer and the isolation layer were bonded together using 3 M Scotch double-sided tape. The hydrophobic isolation layer allows efficient heat transfer from the heating resistor to the SMPs, and in the meanwhile, eliminates any fluid leakage from the channel layer to the heater layer (Fig. 3b).

To carry out an ELISA, proteins always need to be immobilized onto the surfaces of cellulose microfibers of the test zone. We treated the paper test zone through aldehyde functionalization (Fig. S5). The test zone was baked at 65 °C and then spotted with 3 μL of KIO_4_ aqueous solution (0.031 M and pH = 5) for oxidation, and the spotting was repeated every 5 min for 2 h. After oxidization, 10 μL of deionized water (diH_2_O) was added for washing away the residual oxide, and the washing process was repeated twice. Finally, the paper was dried in a desiccator for at least 12 h before use. The aldehyde groups created on the cellulose skeleton of the paper test zone can covalently immobilize proteins containing amino groups through the Schiff-base linkage^41^. The effectiveness of the oxidization process was confirmed by Fourier-transform infrared spectroscopy (FTIR). From the FTIR spectrum of the KIO_4_-modifed paper surface (Fig. S6), the characteristic absorption band of aldehyde group on the paper surface appeared at 1726 cm^−1^ due to the stretching vibration of the C=O double bond^42^.

Before each assay, reagent solutions were added to their corresponding storage zones of a µPAD, and dried at room temperature for 10 min. To determine the amount of reagent that needs to be stored in the test zone, we tested the reagent transfer efficiency from the storage zone to the test zone using fluorophore-tagged antibody as a tracking reagent. Note all the storage zones and their downstream channels connecting the test zone are identical. 3 μL of FITC-conjugated rabbit IgG antibody (anti-rabbit IgG; 0.1 mg/mL) was transferred from the storage zone to the test zone, and the continuous transfer flow of the PBS buffer lasted for 45 s. By measuring the fluorescence intensity of the storage zone before and after the transfer, we found that ~10% of the stored FITC-conjugated anti-rabbit IgG was eventually delivered to the test zone. Thus, the concentrations of the reagent solutions added to the storage zone was determined to be ten times the regular concentrations of the reagents used in a previously reported ELISA protocol on µPAD^3^.

Finally, a plastic lamination layer, which was laser-cut out of 0.127-mm-thick plastic film (Thermal Laminating Pouches TP5903-20, 3M), was attached to the top surface of the channel layer using an impulse heat sealer (PFS-100, Goplus). Four lamination lines (Fig. 3a) were applied to the peripheral areas of the channel layer to bond the plastic lamination layer and the channel layer. This line bonding ensures that the thermal lamination does not impact the activity of the stored reagents.

## Conclusion

We developed a novel SMP-actuated, controllable fluid valve for fluid manipulation on a μPAD, which enabled a paper-based microfluidic platform for automated, multi-step ELISA. The merits of this new platform are summarized as follows. (i) The first SMP-actuated, on-chip paper valve for automatic fluid manipulation: The valve is activated by localized Joule heating, and its relatively small footprint allows the integration of multiple valves onto a single µPAD to run multi-step assays. Thanks to the dual-state operation of the SMP actuator, the valve can be turned on and off using the sample heating resistor, and the length of its on-state period can be readily regulated. (ii) A new single-layer µPAD design with several SMP-actuated valves for automated multi-step ELISA. The µPAD design leverages valve-regulated fluid transfer to enable automated operation of multiple assay steps involving reagent addition, incubation, washing, and signal amplification. (iii) A sensitive light-transmittance-based mechanism for colorimetric signal detection on a µPAD. We demonstrated that, for the same readout signal on our single-layer µPAD, our colorimetric reader, which measured the light transmittance of the paper test zone, provided better LOD and COD of the calibration curve than a desktop scanner and a cellphone camera. In addition, our signal measurement setup only employed a pair of LED and RGB sensor mounted on the colorimetric reader, which is a more cost-efficient and integrated solution than a separate measurement device. (iv) A novel light-transmittance-based mechanism for valve malfunction detection. The operation success rate of the SMP-actuated valves was determined to be 97%. Based on optical measurement of the wetting condition of the paper test zone at different operation steps, this mechanism reliably monitored the operations of all the on-chip valves and flagged any valve malfunction to completely eliminate the malfunction-induced measurement errors. (v) An integrated, user-friendly design of the diagnostic platform, including the µPAD and the colorimetric reader, to realize automated ELISA operation, valve malfunction monitoring, final data collection, and wireless data transmission, if desired.

Although multi-step ELISA was demonstrated on our platform, the designs of the μPAD and the entire platform can be readily extended to implement other types of multi-step diagnostic assays in a SIAO fashion, and are suitable for rapid diagnostic tests at the POC or in any settings where sophisticated equipment and skilled personnel are not available. Examples of other colorimetric assays our platform can be applied to include: glucose^2^, protein^43^, uric acid^44^, lactate^45^, pH^46^, and pathogenic bacteria^47,48^.

## Supplementary information

Supplementary information.

Video S1. Loading a μPAD

Video S2. Operating a μPAD
