# Supplementary material for: A paper-based microfluidic platform with shape-memory-polymer-actuated fluid valves for automated multi-step immunoassays
Source: Microsyst Nanoeng. 2019 Sep 23;5:50. doi: 10.1038/s41378-019-0091-0 (PMC6799814; doi:10.1038/s41378-019-0091-0)
Supplement: Supplementary file 1 — Supplementary information. [file 41378_2019_91_MOESM1_ESM.docx]

A paper-based microfluidic platform with shape-memory-polymer-actuated fluid valves for automated multi-step immunoassays

*Hao Fu^1,2^, Pengfei Song^1,2,3^, Qiyang Wu^1,2^, Chen Zhao^1^, Peng Pan^1,2^, Xiao Li^2^****^,^****^6^, Nicole Y. K. Li-Jessen^4^, and Xinyu Liu^1,5*^*

^1^Department of Mechanical and Industrial Engineering, University of Toronto, Toronto, ON M5S 3G8, Canada

^2^Department of Mechanical Engineering, McGill University, Montreal, QC H3A 0C3, Canada

^3^Department of Electrical and Electronic Engineering, Xi’an Jiaotong-Liverpool University, Suzhou, Jiangsu 215123, China ^4^School of Communication Sciences and Disorders, McGill University, Montreal, QC H3A 1G1, Canada

^5^Institute of Biomaterials and Biomedical Engineering, University of Toronto, Toronto, ON M5S 3G9, Canada

^6^Current address: Department of Chemistry, Stanford University, Stanford, CA, 94305, USA

^*^Corresponding author: Xinyu Liu (xyliu@mie.utoronto.ca)

**Fig. S1** Graphic user interface (GUI) of the smartphone application (APP) for the Bluetooth data communication with the platform. Using the APP, a user can control the platform through Bluetooth to start an assay and receive the test result after the assay is finished. R, G, B, and GS represent the red, green, blue intensity values, and the mean grayscale intensity, respectively.


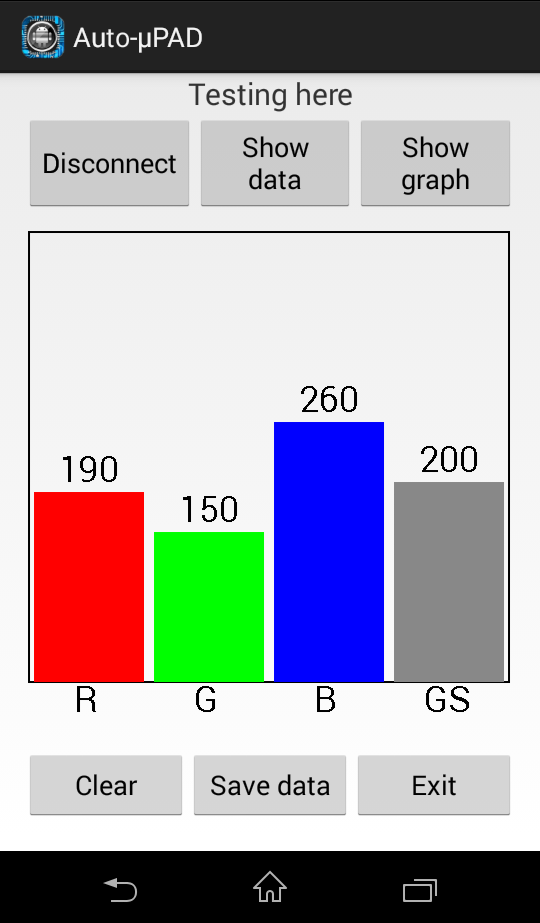


**Fig. S2** Optimization of the reagent transfer time and the washing time. (a) Normalized fluorescence intensity of FITC-conjugated rabbit IgG antibody transferred to the test zone of a µPAD after certain periods of reagent transfer time (n = 5). (b) Normalized fluorescence intensity of residual FITC-conjugated rabbit IgG antibody in the test zone after certain periods of washing time by PBS. All the results shown in (a) and (b) were normalized to the maximum fluorescence intensity among all the measurement data measured from the transferring and washing processes.


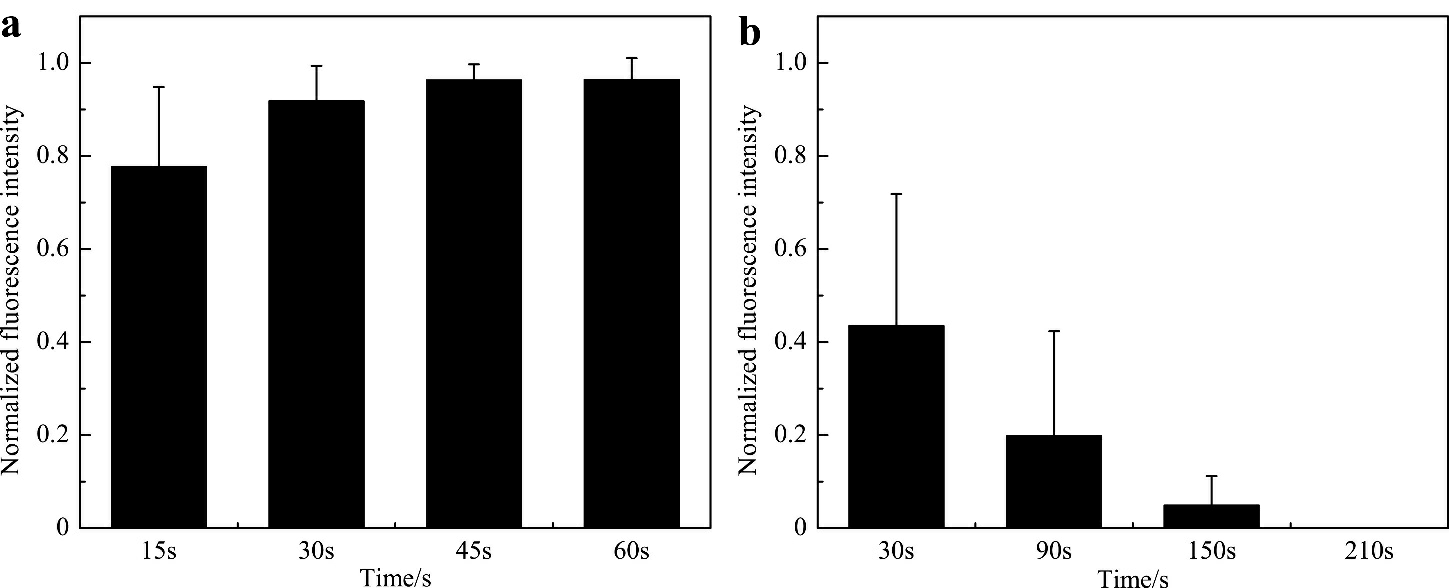


**Fig. S3** The signal-to-noise ratios (SNRs) of different quantification methods including RGB color sensor (black), scanner (red), and camera (blue) based on direct ELISAs for rabbit IgG in 10-fold dilutions (6.7 pM – 6.7 μM) on the platform (n = 5). The dash line indicates the average value of each quantification method.


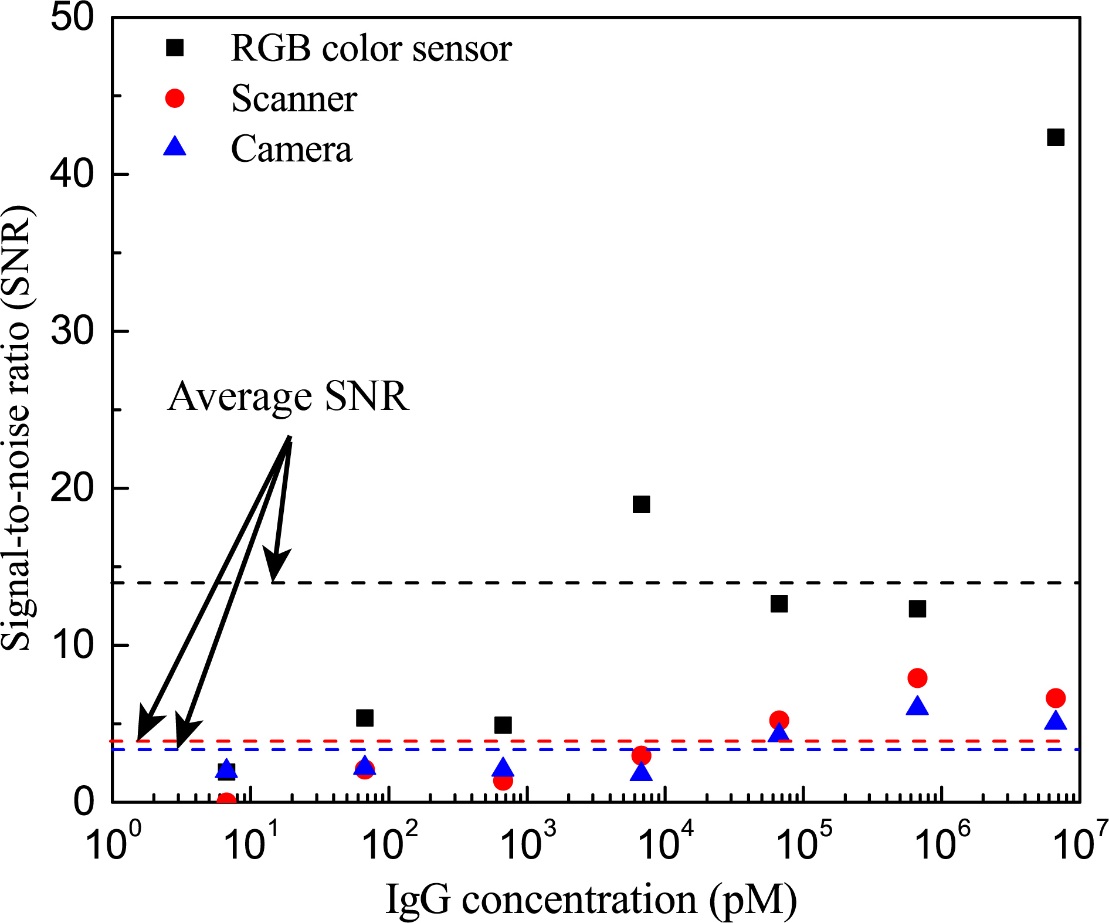


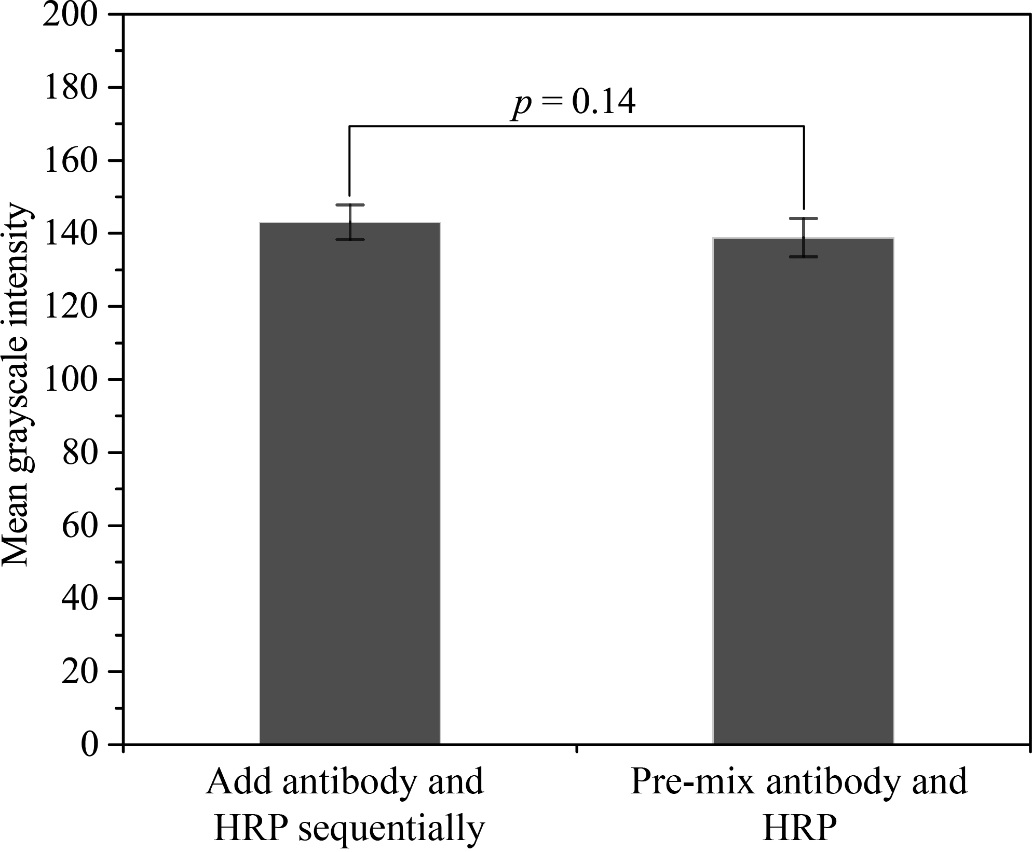


**Fig. S4** The mean grayscale intensities of the sandwich ELISA results for TNF-α in PBS by using two slightly different protocols: (i) adding secondary antibody and HRP streptavidin sequentially to the test zone, and (ii) pre-mixing of secondary antibody and HRP streptavidin and then added the mixture to the test zone (our final protocol adopted). No significant difference (student t-test, *p* = 0.132; n = 7) was observed between the results based on the two protocols.

**Fig. S5** Surface chemical modification of the paper test zone for covalent binding of proteins. (a) Potassium periodate (pH = 5, 0.031 M KIO_4_) is utilized to oxidize the C2-C3 vicinal hydroxyl groups into dialdehyde. (b) Amino groups of the residues (i.e. lysine, asparagine, arginine, or alutamine) in proteins can be covalently bound to the aldehyde groups of modified cellulose surface on the test zone via Schiff base linkage.


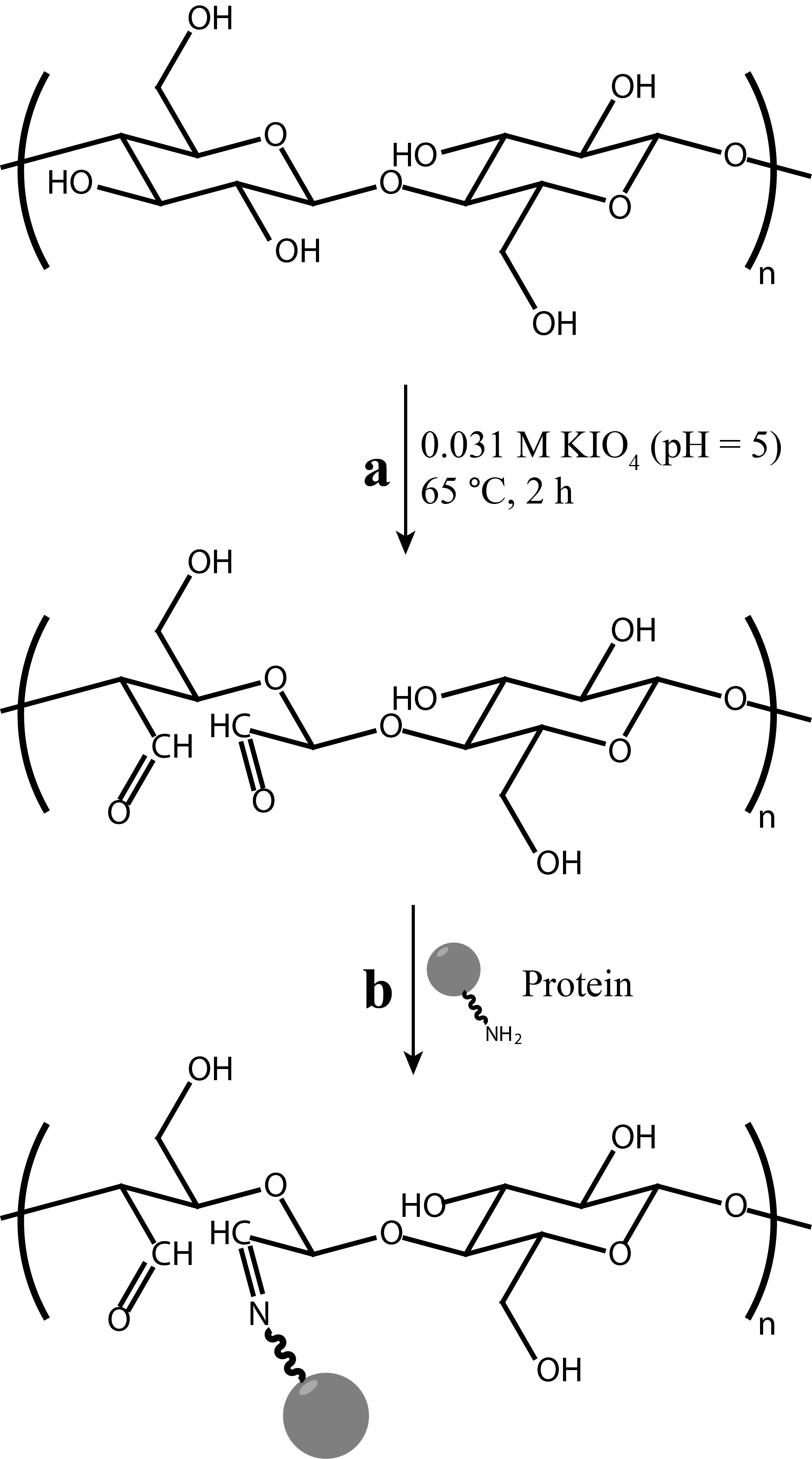


**Fig. S6** Characterization of biofunctionalized test zone using Fourier-transform infrared spectroscopy (FTIR). (a) FTIR spectra of unmodified cellulose paper. (b) FTIR spectra of KIO_4_ modified cellulose paper. The absorption peak at 1726cm^-1^ showed an evidence of aldehyde groups due to the stretching vibration of the C=O double bond.


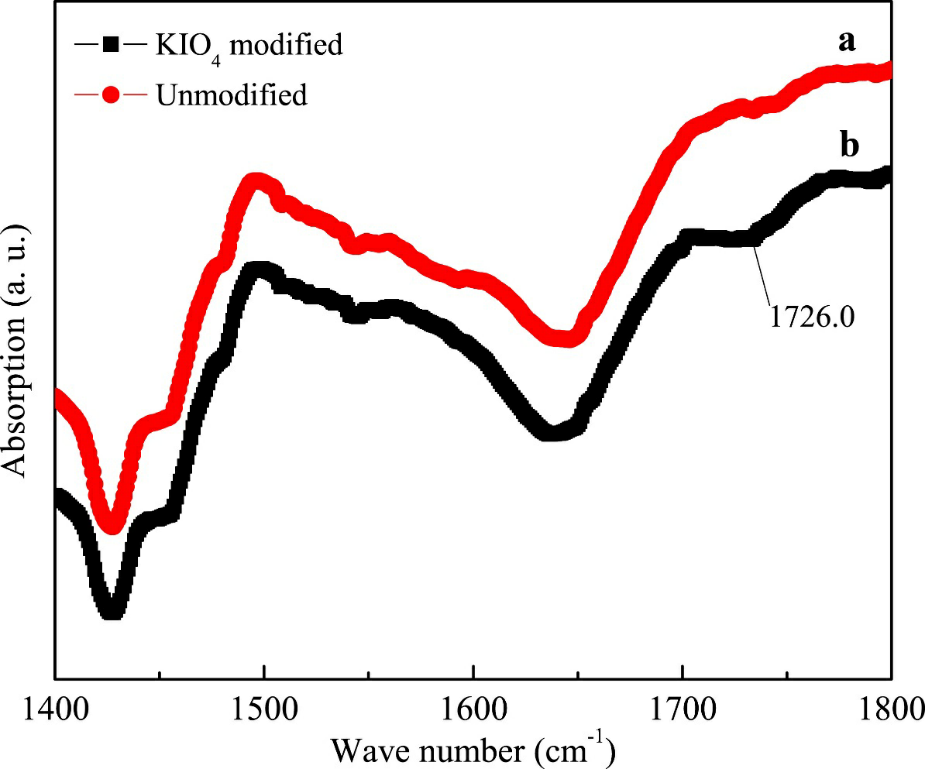


**Table S1.** Comparison of valving performance of three SMPs when SMP-actuated valves (n = 15) were on the platform. Time of activation #1 was the heating time of a SMP to its temporary flat shape. Time of activation #2 was the heating time of the SMP returning to its permanent curved shape. A successful valve activation was counted only with success in both activation #1 and activation #2.

| SMP-actuated valves | Time of  activation #1  (s) | Time of  activation #2  (s) | Success rate  (%) |
| --- | --- | --- | --- |
| 3/8 IN×4 FT BLK  (thickness = 0.64 mm) | 13.3 ± 1.7 | 14.7 ± 2.7 | 33 |
| RNF-100 1”×4’ BLK  (thickness = 0.89 mm) | 22.7 ± 3.7 | 24.4 ± 5.3 | 93 |
| RNF-100 2”×4’ BLK  (thickness = 1.14 mm) | 30.9 ± 4.6 | 71.5 ± 19.6 | 73 |

$\begin{aligned} I=\frac{I_{max}\left[ L \right]^{n}}{\left[ L \right]^{n}+ {{[L}_{50}]}^{n}}\#\left( E1 \right) \end{aligned}$

where *I* is the obtained signal in mean grayscale intensity, *I_max_* is the maximum intensity, [*L*] is the ligand concentration, [*L_50_*] is the ligand concentration corresponding to 50% the binding sites occupied, and *n* is the Hill coefficient.
